# Misinformation and women's health: Rebuilding trust in evidence‐based care

**DOI:** 10.1002/ijgo.70753

**Published:** 2025-12-13

**Authors:** Zainab Al‐Jawahiri

**Affiliations:** ^1^ The Warrell Unit Saint Mary's Hospital, Manchester University NHS Foundation Trust Manchester UK

**Keywords:** clinician communication, evidence‐based medicine, health literacy, misinformation, public health

In September 2025, US president Donald Trump claimed publicly that acetaminophen (sold as Tylenol in the US) use during pregnancy is associated with a “*very increased risk of autism*.”^1^ This assertion, entirely unsupported by current scientific evidence,^2^ spread rapidly across social media and traditional news outlets. Despite swift responses from the medical community clearly rejecting this claim,^3^ the episode exposed the fragility of public trust in health guidance and highlighted an urgent and evolving challenge—the epidemic of medical misinformation. Addressing this issue is not merely a matter of correcting falsehoods, but an essential component of safeguarding public health, and preserving trust in medicine.

Misinformation in medicine is far from a modern problem. In the 1800s, the introduction of the smallpox vaccine provoked immediate public opposition, with conspiracy theories circulating that vaccinated children would develop “cow‐like” deformities.^4^ In the 1950s, concerns about chemicals and state control drove opposition to water fluoridation in the UK, driven by groups like the British Housewives League.^5^ However, what differentiates contemporary medical misinformation is its unprecedented speed and reach. Digital platforms amplify emotionally charged falsehoods, privileging engagement and sensationalism over accuracy. A 2022 survey of over 6200 social media users found that two‐thirds of respondents reported difficulty discerning true from false health information online, and those struggling most were significantly more likely to use social media content when making health decisions.^6^

Historically, women's health has been underrepresented in biomedical research, leading to a perception that mainstream medicine inadequately addresses female‐specific concerns. Public health disasters such as the thalidomide scandal profoundly undermined confidence in mainstream women's healthcare. Consequently many women turn to social media, online influencers, and alternative practitioners for guidance, particularly after dismissive clinical encounters.^7^ While these platforms can promote peer support and enhance health literacy, they can also act as vectors for misinformation by—amplifying unverified claims and potentially undermining trust in evidence‐based care. For example, a recent British study revealed that some women stop using the contraceptive pill due to negative psychological side‐effects influenced by false content found on social media.^8^ The intersection of structural inequities in research and clinical practice, combined with the pervasiveness and provocativeness of digital misinformation, creates a fertile ground for false narratives to flourish especially within our speciality.

Clinicians often assume that simply presenting accurate information will correct misinformation. Yet research demonstrate that facts alone rarely change beliefs.^9^ Misleading claims and conspiracy theories frequently satisfy psychological needs by offering certainty, control, or belonging in uncertain circumstances. Cognitive biases, such as confirmation bias, further entrench belief in misinformation. In obstetric settings, non‐conventional healthcare approaches often align with protective maternal instincts and cultural values emphasizing naturalness and caution.

So how should healthcare professionals respond when patients hold beliefs shaped by misinformation? Evidence from communication science suggests several practical strategies.^10^ Empathic engagement should precede correction by acknowledging intent (“*You want to keep your baby safe—that's understandable*”), to reduce defensiveness and preserve rapport. Techniques such as teach‐back, where patients are asked to articulate their understanding of the information, allow clinicians to identify misconceptions without confrontation and support informed decision making (“*Can you tell me what you read or saw, and what makes it convincing to you?*”). Transparency about uncertainty underscores honesty in the consultation (“*We can't say with absolute 100% certainty—no study can do that*”). Embed correct information within context: lead with accurate guidance, explain the misinformation, and conclude with truth (“*Acetaminophen remains the recommended pain relief in pregnancy. Some studies found weak associations with neurodevelopment, but none prove causation. Used correctly, it is safe*”). Be open to revisiting the topic in subsequent consultations to foster reflection and ongoing dialogue (“*Last time we talked about that Tylenol claim—do you have any more questions about it?*”).

Beyond individual encounters, proactive “pre‐bunking”—anticipating false claims and addressing them before patients encounter them—is highly effective.^11^ This can be integrated into antenatal education, preoperative counseling, and written materials, equipping patients to recognize and resist misleading claims.

Ethical considerations are central in these consultations. Patients have the right to refuse interventions, even if deemed unwise, but clinicians must ensure refusals are informed, voluntary, and free from coercion. Empathy and open dialogue are professional obligations, not optional courtesies; whereas derision or judgment will erode trust and undermine the therapeutic relationship.

Addressing misinformation requires systemic action. Healthcare institutions should implement consistent messaging and provide accessible patient resources responsive to changing trends. Medical education must prepare clinicians to recognize cognitive biases, communicate uncertainty effectively, and respond constructively to misinformation both within and beyond the consultation room.

The healthcare community bears the responsibility to communicate with accuracy, clarity, empathy, and respect. In today's viral information ecosystem, evidence most effectively influences understanding and decision making when delivered in a manner that is precise, professionally responsible, and compelling enough to be heard amidst the noise.

## AUTHOR CONTRIBUTIONS

ZA‐J: Conceptualization, writing, final approval, and accountability.

## FUNDING INFORMATION

The author has nothing to report.

## CONFLICT OF INTEREST STATEMENT

The author has nothing to report.

## ETHICS STATEMENT

Not applicable.

## INFORMED CONSENT

Not applicable.

## Data Availability

Data sharing is not applicable to this article as no new data were created or analyzed in this study.
